# Reporting the Reliability of Accelerometer Data with and without Missing Values

**DOI:** 10.1371/journal.pone.0114402

**Published:** 2014-12-05

**Authors:** Eric E. Wickel

**Affiliations:** Exercise and Sports Science, University of Tulsa, Tulsa, OK, United States of America; University of Louisville, United States of America

## Abstract

**Objectives:**

Participants with complete accelerometer data often represent a low proportion of the total sample and, in some cases, may be distinguishable from participants with incomplete data. Because traditional reliability methods characterize the consistency of complete data, little is known about reliability properties for an entire sample. This study employed Generalizability theory to report an index of reliability characterizing complete (7 days) and observable (1 to 7 days) accelerometer data.

**Design:**

Cross-sectional.

**Methods:**

Accelerometer data from the Study of Early Child Care and Youth Development were analyzed in this study. Missing value analyses were conducted to describe the pattern and mechanism of missing data. Generalizability coefficients were derived from variance components to report reliability parameters for complete data and also for the entire observable sample. Analyses were conducted separately by age (9, 11, 12, and 15 yrs) and daily wear time criteria (6, 8, 10, and 12 hrs).

**Results:**

Participants with complete data were limited (<34%) and, most often, data were not considered to be missing completely at random. Across conditions, reliability coefficients for complete data were between 0.74 and 0.87. Relatively lower reliability properties were found across all observable data, ranging from 0.52 to 0.67. Sample variability increased with longer wear time criteria, but decreased with advanced age.

**Conclusions:**

A reliability coefficient that includes all participants, not just those with complete data, provides a global perspective of reliability that could be used to further understand group level associations between activity and health outcomes.

## Introduction

Wearable monitors overcome many of the limitations of self-report methods and are now regularly used to objectively assess free-living physical activity (PA) in children and adolescents. Accelerometers, such as the Actigraph, are a common type of wearable monitor that, in contrast to other objective tools (e.g., pedometers), are capable of characterizing the frequency, intensity, duration, and time of daily PA. Despite the accelerometer's appeal as a field-based assessment tool, several data management and processing challenges exist [1].

One specific challenge accelerometer end-users must contend with involves missing data. Missing data are inherent to nearly all free-living accelerometer studies and can exist as repeated episodes within a day or across entire day(s). Based on Rubin's taxonomy [2], missing data are classified as missing at random (MAR), missing completely at random (MCAR), or not missing at random (NMAR). In most cases, end-users do not report the mechanism of data missingness, but rather address critical decisions about the identification of missing data (i.e., non-wear time) as well as the consequences associated with the quantity of missing data. Automated algorithms, such as those used in analyzing accelerometer data from NHANES [3], are routinely employed to identify and remove non-wear time throughout daily accelerometer records, thus allowing wear time estimates to be reported for a 24-hr period or a pre-defined portion of the day (e.g. 7:00 to 22:00). Decisions based on wear time are then made to remove individual monitoring days (e.g., days with <10 hrs) or entire participant records (e.g., participants with <4 valid days). This general approach is commonly employed during data treatment to produce estimates of total daily activity, as well as daily proportions of activity intensities, but can result in a loss of data.

Several techniques have been used to recover missing data from individual monitoring days and some of these methods have yielded unbiased estimates of PA [4]–[8]. In comparison, relatively less is available regarding missing data and reliability estimates, which is surprising given the role reliability coefficients play in PA research. As noted by Brennan [9], reliability is a characteristic of scores, and in PA research this basic definition describes the variability across repeated days. Routinely, reliability is reported using standard approaches like the intraclass correlation coefficient (ICC). Many statistical programs readily calculate the ICC; however, in doing so a considerable portion of participants are often removed via list-wise deletion which in turn decreases power and external validity. Although the percentage of participants with complete accelerometer data (complete data may be defined as having ≥ 10 hrs of wear time across 7 consecutive days) likely varies across studies, pooled data from the International Children Accelerometer Database [10] indicate few youth (ages 9 to 18 yrs) have complete data during a standard 7-day monitoring protocol. This robust finding suggests ICC-derived reliability coefficients for moderate-to-vigorous physical activity (MVPA) reported in many, but not all, accelerometer studies are derived from a relatively low percentage of available participants when imputation or multilevel regression models are not employed. To expand our perspective of reliability, alternative approaches that account for all observable (non-imputed) data should be explored.

Cronbach and colleagues [11] introduced generalizability (G) theory as an approach to examine reliability. In contrast to classical measurement models where the error term is singular, G theory can be used to partition and quantify variance according to multiple sources of error (G study) so an investigator can make informed decisions regarding the design needed to maximize reliability (Decision (D) study) [12]. Within the G theory framework, techniques to report a reliability coefficient for unbalanced designs (i.e., studies with missing data (1 ≤ number of acceptable days (*n_d_*) ≤ 7)) exist but have yet to be explored in PA research. Reporting a reliability coefficient that includes all participants, not just those with complete data, provides a global perspective of reliability that may be useful in understanding group-level associations between MVPA and health outcomes.

To advance reliability research, the current study details the application of G theory to report an index of reliability using complete (*n_d_*  =  7) and observable (1 ≤ *n_d_* ≤ 7) accelerometer data. All analyses were conducted using daily levels of MVPA, which were available from a large prospective study of youth at 9, 11, 12, and 15 yrs of age.

## Methods

Accelerometer data from the Study of Early Child Care and Youth Development were analyzed to address the study objective. The original monitoring protocol was approved by each participating university's ethics committee (University of Arkansas; University of California; University of Kansas; University of New Hampshire/Wellesley; Pennsylvania State University/University of Pittsburgh; Temple University; University of Virginia; University of Washington; Western Carolina Center; and University of Wisconsin) and written consent was obtained from each participant. Details regarding the enrollment procedure and research protocol are available from the study's website (http://www.nichd.nih.gov/research/supported/seccyd/pages/overview.aspx). Accelerometer data were collected across a 7-day monitoring period using 1-minute epochs at mean ages of 9, 11, 12, and 15 yrs. ActiLife software (version 6.4.3) was used to detect and remove daily non-wear intervals between the hours of 7:00 and 22:00. Similar to other studies [3], non-wear periods included intervals of at least 60 consecutive minutes of zero activity counts, allowing for 2 minutes of counts between 0 and 100. Epochs exceeding 20,000 counts/min were reset to zero. Daily wear time was determined by removing daily non-wear periods. At each mean age, four separate data sets were created using minimum daily time requirements of 6, 8, 10, and 12 hrs. Accelerometer data were then interpreted in a manner consistent with the approach used in the International Children Accelerometer Database [10], where the amount of MVPA (mins/day) was determined using a threshold of 3000 counts/min. A total of 1082 youth were enrolled in the accelerometer protocol at 9 yrs. Age-related trends in activity have been previously reported with these data [13]; however, the objective of the current study was not addressed.

Descriptive analyses were conducted for accelerometer wear time and MVPA by age and wear time criteria. Missing value analyses were conducted to report the proportion of missing data by day of the week and Little's chi-square statistic was used to report the mechanism of missingness. The null hypothesis for Little's chi-square test states that the data are MCAR; therefore, p-values <0.05 were considered significant and under this circumstance the missing data would be MAR or NMAR. Several reviews of MAR, MCAR, and NMAR exist [14], [15], but a brief interpretation is provided. Missing data are likely to be MAR [16], and under this mechanism the pattern of missingness is systematically related to some observed characteristic. In this situation, it is assumed that the actual variables where data are missing are not the cause of the incomplete data. MCAR is a sub-category of MAR [17], but comparatively more stringent, and assumes that missing data are unrelated to the variables being studied. In this context, individuals with missing data represent a simple random sample of the full sample (i.e., individuals with complete data are indistinguishable from those with incomplete data). Under the third mechanism (NMAR), the pattern of missing data is related to unobserved characteristic(s). Of the three missing data mechanisms, only MCAR can be empirically tested because MAR and NMAR are dependent upon unobserved data. Descriptive and missing value analyses were conducted using SPSS v20.

To address the study's primary objective, reliability coefficients using complete (*n_d_*  =  7) and observable (1 ≤ *n_d_* ≤ 7) accelerometer data were compared using G theory methods. Although G theory has been described in the literature [18], [19], few studies have applied this approach to PA research [12], [20]–[24]. Following the framework outlined by Brennan [18], the current study employed a single facet (participant × day) design with missing data, where variance component estimates were derived using analogous T terms for the object of measurement (participants (*ô^2^p*)), the facet (day (*ô^2^d*)), and the interaction term which is confounded with unsystematic or unmeasured error (*ô^2^ pd*). Derived variance components were then used to calculate two types of error (absolute (*ô^2^Δ*) and relative (*ô^2^δ*)). Absolute, or criterion-referenced, error is the error involved in using a participant's mean score as an estimate of their universe score (i.e., *ô^2^Δ*  =  (*ô^2^ d*/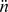 *_d_*) + (*ô^2^ pd*/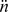 *_d_*)), whereby 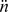 *_d_* is the harmonic mean of 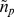 (i.e., the number of days with acceptable data from each participant) [18] and is derived using Equation 1. 
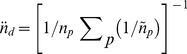
(Equation)


In contrast, relative error is associated with norm-referenced interpretations of measurement and equals the variance of the observed mean score for participants (*S^2^ p*) minus participant variance (*ô^2^δ*  =  *S^2^ p* – *ô^2^ p*) (Equation 2). 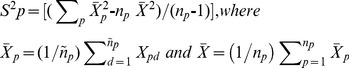
(Equation)


A complex issue arises in the calculation of *S^2^ p* because the mean score for each participant is based on a different number of acceptable days, ranging from 1 to 7.

Variance components obtained from the unbalanced design were used in the D study to derive a reliability coefficient that characterized complete data (*n_d_*  =  7) (*Ep^2^_Complete_*  =  *ô^2^ p*/[*ô^2^ p* + (*ô^2^ pd*/*n_d_*)]) and, specific to this study, a separate coefficient that characterized all observable data (1≤ *n_d_* ≤ 7) (*Ep^2^_Observed_*  =  *ô^2^ p*/*S^2^ p*). Coefficients range from 0 to 1. In PA research, reliability coefficients ≥ 0.80 are desirable. Standard error of the mean (SEM), which provides an indication of the uncertainty associated with each measure, was calculated for each condition by taking the square root of the absolute error term. The SEM is expressed in the same metric unit of measurement and represents a 68% CI for the participant's universe score. Variance components, error estimates, and reliability coefficients were derived using EXCEL macros created by the corresponding author (see Tables S1 and S2 for a detailed description of the G theory calculations).

## Results

Missing data characteristics are reported in Table 1. As expected, the percentage of youth with complete accelerometer data decreased as daily wear time criteria increased from 6 to 12 hrs. In general, the proportion of missing data was similar across weekdays, but relatively higher during the weekend. Under most conditions the null hypothesis for Little's chi-square test was rejected, suggesting the data were not MCAR and that participants with complete data were distinguishable from those with incomplete data. Mean weekly comparisons for accelerometer wear time (hrs/day) and MVPA (mins/day) between complete and incomplete data support this conclusion across most conditions (Table 2). Post-hoc analyses were conducted separately for weekdays and weekend days to examine each MCAR condition reported in Table 1. For each MCAR condition, non-distinguishable mean values were observed at 9 yrs (weekend wear time and MVPA), 11 yrs (weekend wear time), 12 yrs (weekend wear time and MVPA; weekday MVPA), and 15 yrs (weekend wear time and MVPA; weekday MVPA) (data not shown). As shown in Table 2, mean wear time typically exceeded 12 hrs and progressively increased as longer wear time criteria were employed. Among the combined sample (1 ≤ n_d_ ≤ 7), the mean absolute difference in MVPA between 6- and 12-hr criteria approximated 3 mins, and the absolute percent error (APE) for MVPA increased with advancing mean age (9 yrs: 11.2%; 11 yrs: 12.9%; 12 yrs: 15.8%; and 15 yrs: 20.4%). APE estimates were derived using MVPA levels from the 6- and 12-hr wear time criteria (((|MVPA_6 hrs_ – MVPA_12 hrs_|)/MVPA_12 hrs_) × 100).

**Table 1:** Missing data pattern and mechanism by age and wear time criteria.

		% of days with missing data							
Condition	*n_p_* (% with 7 days)	Mon	Tue	Wed	Thu	Fri	Sat	Sun	Missing data mechanism
9 years									
6 hrs	807 (34)	19	16	20	17	21	27	23	MAR/NMAR a
8 hrs	801 (29)	21	17	21	18	22	32	28	MAR/NMAR a
10 hrs	788 (20)	24	20	25	22	25	40	42	MAR/NMAR a
12 hrs	762 (7)	36	33	34	33	34	57	65	MCAR
11 years									
6 hrs	857 (33)	17	18	21	20	19	28	28	MAR/NMAR a
8 hrs	851 (26)	19	19	22	22	22	34	34	MAR/NMAR a
10 hrs	834 (16)	23	22	26	26	28	47	47	MAR/NMAR a
12 hrs	801 (4)	34	36	34	37	38	67	72	MCAR
12 years									
6 hrs	733 (24)	22	27	25	23	25	35	35	MCAR
8 hrs	729 (20)	25	27	26	26	27	43	42	MAR/NMAR a
10 hrs	713 (12)	28	30	31	29	32	52	55	MAR/NMAR a
12 hrs	661 (3)	35	39	38	37	38	69	77	MCAR
15 years									
6 hrs	656 (17)	29	25	30	30	33	50	43	MAR/NMAR a
8 hrs	642 (13)	32	28	30	31	35	56	50	MAR/NMAR a
10 hrs	611 (8)	36	31	33	35	41	66	64	MAR/NMAR a
12 hrs	558 (2)	45	40	39	42	47	81	84	MCAR

MAR, missing at random; MCAR, missing completely at random; NMAR, not missing at random; *n_p_*, number of participants.

a Little's test, p <0.05.

**Table 2:** Mean weekly wear time and MVPA between youth with complete (*n_d_*  =  7) and incomplete (1 ≤ *n_d_* ≤ 6) data.

	Mean (SD) wear time - hours/day			Mean (SD) MVPA - minutes/day		
Condition	Complete	Incomplete	Difference (95% CI)	Complete	Incomplete	Difference (95% CI)
9 years						
6 hrs	12.6 (0.9)	12.2 (1.4)	0.42 (0.26 to 0.58)	27 (11)	32 (15)	−5.4 (−7.3 to −3.6)
8 hrs	12.8 (0.8)	12.4 (1.1)	0.36 (0.22 to 0.50)	27 (12)	32 (15)	−5.1 (−7.1 to −3.2)
10 hrs	13.1 (0.6)	12.8 (0.8)	0.26 (0.15 to 0.38)	27 (12)	33 (15)	−5.4 (−7.6 to −3.1)
12 hrs	13.6 (0.4)	13.4 (0.6)	0.18 (0.06 to 0.31)	30 (11)	34 (16)	−4.3 (−7.6 to −1.0)
11 years						
6 hrs	12.4 (1.1)	12.1 (1.4)	0.29 (0.11 to 0.46)	20 (9)	26 (13)	−6.3 (−7.8 to −4.9)
8 hrs	12.7 (0.8)	12.3 (1.2)	0.39 (0.24 to 0.53)	20 (9)	26 (13)	−6.2 (−7.7 to −4.7)
10 hrs	13.2 (0.6)	12.8 (0.9)	0.36 (0.23 to 0.48)	20 (9)	27 (13)	−6.7 (−8.5 to −4.8)
12 hrs	13.7 (0.4)	13.5 (0.6)	0.18 (0.05 to 0.32)	21 (9)	28 (14)	−7.0 (−10.4 to −3.6)
12 years						
6 hrs	12.6 (1.0)	12.0 (1.6)	0.59 (0.39 to 0.79)	16 (8)	20 (11)	−4.3 (−5.8 to −2.9)
8 hrs	12.8 (0.9)	12.3 (1.3)	0.47 (0.29 to 0.65)	17 (8)	21 (11)	−3.8 (−5.4 to −2.2)
10 hrs	13.2 (0.7)	12.9 (1.0)	0.33 (0.16 to 0.49)	18 (9)	21 (12)	−3.8 (−6.0 to −1.7)
12 hrs	13.9 (0.4)	13.6 (0.6)	0.28 (0.10 to 0.46)	20 (8)	23 (13)	−3.3 (−6.9 to 0.3) a
15 years						
6 hrs	12.5 (1.2)	11.8 (1.8)	0.69 (0.41 to 0.97)	9 (6)	12 (9)	−3.0 (−4.4 to −1.6)
8 hrs	12.8 (1.1)	12.3 (1.5)	0.50 (0.23 to 0.77)	9 (7)	13 (10)	−3.2 (−4.8 to −1.6)
10 hrs	13.5 (0.6)	13.0 (1.1)	0.53 (0.33 to 0.74)	9 (7)	13 (10)	−3.7 (−5.8 to −1.7)
12 hrs	14.3 (0.4)	13.8 (0.7)	0.45 (0.17 to 0.72)	8 (5)	14 (11)	−6.8 (−10.3 to −3.2)

MVPA, moderate-to-vigorous physical activity; *n_d_*, number of days.

a Mean difference in MVPA between complete and incomplete data was not significant (p> 0.05). Remaining mean difference values (wear time and MVPA) were significant.

Total variance in MVPA increased with longer wear time criteria and decreased with increasing age. In general, total variance was distributed in a similar manner across conditions (p × d interaction term > participant term > day term). The relatively large contribution of variance from the p × d interaction term (ranging from 47 to 68%) reflects the wide range of individual variability in daily free-living MVPA levels across days, but also includes unexamined error. The participant term reflects inter-individual variation and explained a majority of the remaining error (ranging from 27 to 47%). Ideally, the participant term would account for the largest proportion of error because it represents true score variance. In comparison, a relatively low amount of variation was attributable to the day term (1 to 9%), suggesting little variability in MVPA across monitoring days. Variance components and error estimates of MVPA (*ô^2^δ* and *S^2^ p*) are reported in Table 3. The findings show an increase in sample variability with longer wear time criteria, but also reveal a trend toward sample homogeneity with increasing age. The harmonic mean of 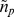, reported as 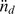, reveals the number of acceptable days across participants and is included in the absolute error variance calculation. This value decreased with increasing age and wear time criteria (6 to 12 hrs) (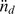 at 9 yrs (4.7, 4.5, 4.1, and 3.1 days); 11 yrs (4.6, 4.4, 3.9, and 2.9 days); 12 yrs (4.0, 3.6, 3.2, and 2.7 days); and 15 yrs (3.4, 3.3, 2.9, and 2.3 days). SEM values ranged from 5 to 9 mins across conditions.

**Table 3:** Variance components, error estimates and generalizability coefficients of MVPA by age and wear time criteria.

	Variance components			Error estimates		Generalizability coefficients	
Condition	*ô* 2 *p*	*ô* 2 *d*	*ô* 2 *pd*	*ô* 2 *δ*	*S* 2 *p*	*Ep* 2 *_Complete_* 1	*Ep* 2 *_Observed_* 2
9 years							
6 hrs	131.2	12.3	221.3	65.7	196.9	0.81	0.67
8 hrs	138.1	11.7	223.1	70.1	208.1	0.81	0.66
10 hrs	143.4	10.5	221.8	75.7	219.1	0.82	0.65
12 hrs	154.1	8.1	237.1	101.0	255.2	0.82	0.60
11 years							
6 hrs	84.3	8.6	187.5	58.8	143.0	0.76	0.59
8 hrs	87.0	6.8	186.3	62.1	149.1	0.77	0.58
10 hrs	93.5	5.5	188.5	70.9	164.3	0.78	0.57
12 hrs	117.1	3.4	202.1	84.6	201.7	0.80	0.58
12 years							
6 hrs	57.9	11.0	145.3	44.9	102.8	0.74	0.56
8 hrs	61.8	9.7	148.6	49.9	111.6	0.74	0.55
10 hrs	75.8	7.0	157.9	64.7	140.5	0.77	0.54
12 hrs	84.1	4.6	161.0	77.9	162.0	0.79	0.52
15 years							
6 hrs	48.4	12.1	73.3	30.0	78.3	0.82	0.62
8 hrs	52.7	12.8	77.8	35.2	87.9	0.83	0.60
10 hrs	59.8	12.4	78.1	35.8	95.6	0.84	0.63
12 hrs	77.5	10.5	78.5	42.9	120.4	0.87	0.64

1 Generalizability coefficient derived using complete data (*n_d_*  =  7).

2 Generalizability coefficient derived using observed data (1 ≤ *n_d_* ≤ 7).

*ô^2^ p*, variance component for participant; *ô^2^ d*, variance component for day; *ô^2^ pd*, variance component for participant × day interaction; *ô^2^δ*, relative error variance; *S^2^ p*, observed participant mean score variance.

Reliability methods were expected to produce different coefficients *a priori* given that each coefficient (*Ep^2^_Complete_* and *Ep^2^_Observed_*) characterized unique data. Across age and wear time conditions, reliability coefficients derived from complete data (*Ep^2^_Complete_*; *n_d_*  =  7) were relatively higher compared to coefficients derived from observed data (*Ep^2^_Observed_*; 1≤ *n_d_* ≤ 7) (Table 3). *Ep^2^_Complete_* coefficients ranged from 0.74 to 0.87 and were derived assuming a sample size equal to the original sample (e.g., using 10-hr wear time criteria, *n_p_* = 788 (9 yrs); *n_p_* = 834 (11 yrs), *n_p_* = 713 (12 yrs), and *n_p_* = 611 (15 yrs)) and 7 monitoring days even when variance components were derived with missing data in the G study [18]. In contrast, the global index of reliability (i.e., *Ep^2^_Observed_*) ranged from 0.52 to 0.67. A similar index of reliability would be expected among separate samples given similarities in both sample size and pattern of missing data.

## Discussion

A 7-day monitoring period is recommended for PA research because week and weekend data are included [25], [26].When this standard approach is implemented, data end-users commonly report few participants with complete records. For example, data from NHANES reveal a low proportion (nearly 20%) of youth ages 6 to 19 yrs with seven days of accelerometer data [3]. In the current study, nearly one-third of participants (34%) had a complete 7-day record at 9 yrs and this proportion decreased as age and wear time criteria increased. For the accelerometer end-user, identifying few participants with complete data is concerning because traditional reliability coefficients characterize complete data and participants with complete data (operationally defined across studies as 2 to 7 valid days) may be characteristically different than those with incomplete data [27], [28]. The current study employed G theory to report a reliability coefficient (*Ep^2^_Observed_*) that included all observable data satisfying daily wear time criteria. To date, several perspectives of reliability have been reported in the literature [12], [29], but all focus on complete data. This study addresses a critical gap in the literature and provides accelerometer end-users with an alternative approach to report reliability.

Although data imputation could have been used in this study to increase the proportion of youth with complete accelerometer records, the primary aim was to report a reliability coefficient that characterized all observable (non-imputed) data satisfying daily wear time criteria. Under most age and wear time conditions, the missing data in this study were indistinguishably MAR or NMAR suggesting data imputation may produce biased summary statistics and reliability estimates. Ideally, missing data would be MCAR and represent a simple random proportion of the entire sample. Given the inherent challenges of specifying the pattern of missing data, few studies have directly examined the implications of missing accelerometer data on reliability parameters. In an accelerometer simulation study involving girls, Catellier et al [4] selected those with complete, 7-day accelerometer records and systematically generated datasets with different patterns of missing data (MCAR and NMAR) to compare sample parameters between complete and imputed data sets. Minimal levels of bias in mean daily estimates of MET-mins of MVPA were reported between complete and imputed data when MCAR was assumed; however, a positive bias was reported after imputing missing values that were NMAR. In general, standard deviations for daily MET-mins of MVPA were similar between complete and imputed datasets. Additional youth simulation studies should be conducted to assess the performance of imputation methods. Future studies would likely benefit from the purposeful approach described by Catellier and colleagues to generate missing data characteristic of MCAR and NMAR. Furthermore, the global reliability coefficient described in this study could be reported in future imputation assessment studies (using simulated and non-simulated data) as an additional comparative parameter before and after data imputation occurs. Comparing reliability coefficients in this manner would be novel given that many studies report reliability using complete, rather than observable, data.

Accelerometer data included in this study were processed using ActiLife software to identify complete monitoring days using four daily wear time criteria (6, 8, 10, and 12 hrs) between 7:00 and 22:00. This approach was selected not with the intent to identify a specific wear time threshold, but rather to report the effect of wear time inclusion criteria on factors that influence reliability estimates and levels of MVPA (e.g., sample size and composition). At each mean age, *S^2^ p* and *ô^2^ p* increased as daily wear time criteria became more stringent, indicating greater variability in MVPA within the sample using the 12-hr threshold compared to the more conservative 6-hr threshold. As a general tenet of reliability, increased variability among individuals produces higher reliability estimates [30], and this was seen in the G coefficient with complete data (*Ep^2^_Complete_*) at each mean age. In this study, estimates of *S^2^ p* at a given wear time duration (e.g., 10 hrs) progressively declined with increasing mean age, suggesting youth levels of MVPA may become more homogeneous with age. Certain caveats do exist however when reporting trends in reliability and error estimates. For example, the accelerometer data included in this analysis originated from a prospective study design; however, the sample size and composition at each mean age (and across wear time conditions) varied. Therefore, these trends should be interpreted judiciously given that each age and wear time condition was comprised of unique data.

Relatively small absolute differences in MVPA were found between 6- and 12-hr wear time criteria at each mean age, ranging from 2.8 to 3.7 mins. The relative stability in MVPA estimates found in this study can likely be attributed to the similarities in mean wear time duration. Across conditions, the accelerometer was worn, on average, for nearly 80% of the 15-hr monitoring period. Although daily patterns of MVPA were not specifically examined in the current study, it is quite possible that the additional 1.5 hrs of daily wear time reported using the 12-hr criteria (compared to the 6-hr criteria) occurred during the latter portions of the day, a period when activity levels decline among girls and boys [31]. Varying wear time criteria have been used in PA research to define a valid day, but 10-hrs is generally considered an acceptable duration to capture youth estimates of activity [3], [32]. Empirical evidence to support the 10-hr monitoring duration could be systematically investigated using a semisimulated approach described by Herrmann et al. [33] Among adults, Herrmann et al compared APE estimates in daily PA between semisimulated data sets of varying wear time criteria (10, 11, 12, and 13 hrs/day) to a reference level of 14 hrs/day. It was concluded that time spent in inactivity, light, and moderate activity was nearly 30% less using 10 hrs/day compared to 14 hrs/day. Inspecting levels of bias between wear time levels was not necessarily the focal point of the current study; however, comparatively lower APE estimates of MVPA were found between 6- and 12-hr criteria at each mean age when compared to the APE estimates reported for adults between 10- and 14-hr criteria. Observed differences in APE may likely be attributable to the structural differences in daily accumulation of PA between youth and adults (i.e., timing of PA participation), or to varying mean levels of activity observed during childhood and adolescence. For example, in the present study, APE estimates were comparatively higher at 15 yrs compared to 9 yrs, likely reflecting the variation in mean levels of MVPA between mean ages (MVPA levels nearly 3× higher at 9 yrs compared to 15 yrs) rather than the absolute mean difference between MVPA estimates using 6- and 12-hr criteria (∼ 3 mins). Establishing a standardized wear time duration for youth and adults would be beneficial to minimize bias in error estimates and facilitate group comparisons across studies.

### Conclusion and Future Research Implications

G theory was applied in this study to report an index of reliability using a balanced design with complete data (*n_d_*  =  7) and an unbalanced design using all observable data (1≤ *n_d_* ≤ 7). Reporting a global index of reliability is novel to PA research and may prove useful for investigators interested in reporting parameter characteristics of an entire sample, rather than a sub-sample with complete data. Future research may consider applying a reliability coefficient, like the one described here (*Ep^2^_Observed_*), to Spearman's disattenuation formula [34] to examine the correlation between PA and health-related outcome measures. Future studies should also compare reliability coefficients from an unbalanced design using G theory to reliability coefficients obtained using other multilevel models.

## Supporting Information

Table S1 **Data structure and G theory calculations.**
(DOCX)

Table S2 **Variance component equations and estimates.**
(DOCX)
