# Supplementary material for: Reporting the Reliability of Accelerometer Data with and without Missing Values
Source: PLoS One. 2014 Dec 5;9(12):e114402. doi: 10.1371/journal.pone.0114402 (PMC4257690; doi:10.1371/journal.pone.0114402)
Supplement: Table S1 — Data structure and G theory calculations. (DOCX) [file pone.0114402.s001.docx]

**Table S1.**

**Data structure and interpretation for generalizability theory calculations. Summary values represent actual data at 9 years of age using the 10-hour wear time criteria.**

|  | **Moderate-to-vigorous physical activity (minutes)** | | | | | | |  |  |  |  |  |  |  |  |
| --- | --- | --- | --- | --- | --- | --- | --- | --- | --- | --- | --- | --- | --- | --- | --- |
| **ID** | **Mon** | **Tue** | **Wed** | **Thu** | **Fri** | **Sat** | **Sun** |  | ***ñ_p_*** | ***ñ*** | ***ñ/n_+_*** | **X̄*_p_*** | **X̄** | ***X (ñ_p_)*** | **1/*ñ_p_*** |
| 1 | 12 | 26 | 24 | 11 | 7 | 24 | 8 |  | 7 | 49 | 0.0124 | 16.0 | 256.0 | 1792 | 0.143 |
| 2 | 36 | 10 | 21 | 61 | 17 | 34 | 15 |  | 7 | 49 | 0.0124 | 27.7 | 768.1 | 5377 | 0.143 |
| 3 | 17 | 32 | 9 | 17 | 10 |  | 16 |  | 6 | 36 | 0.0091 | 16.8 | 283.4 | 1700 | 0.167 |
| 4 | 49 | 7 |  | 37 | 58 | 35 | 19 |  | 6 | 36 | 0.0091 | 34.2 | 1167.4 | 7004 | 0.167 |
| 5 | 29 | 41 | 63 | 38 | 25 |  |  |  | 5 | 25 | 0.0063 | 39.2 | 1536.6 | 7683 | 0.200 |
| 6 | 6 | 48 | 30 | 25 |  | 16 |  |  | 5 | 25 | 0.0063 | 25.0 | 625.0 | 3125 | 0.200 |
| 7 | 31 | 38 | 54 |  | 21 |  |  |  | 4 | 16 | 0.0040 | 36.0 | 1296.0 | 5184 | 0.250 |
| 8 | 17 | 26 |  | 10 | 17 |  |  |  | 4 | 16 | 0.0040 | 17.5 | 306.3 | 1225 | 0.250 |
| 9 |  |  | 18 |  | 9 | 9 |  |  | 3 | 9 | 0.0023 | 12.0 | 144.0 | 432 | 0.333 |
| 10 |  | 16 | 22 |  |  |  |  |  | 2 | 4 | 0.0010 | 19.0 | 361.0 | 722 | 0.500 |
| - | - | - | - | - | - | - | - |  | - | - | - | - | - | - | - |
| - | - | - | - | - | - | - | - |  | - | - | - | - | - | - | - |
| 788 |  |  |  | 19 |  |  |  |  | 1 | 1 | 0.0003 | 19.0 | 361.0 | 361 | 1.000 |
|  |  |  |  |  |  |  |  |  | ***∑_p_ ñ_p_*** |  | ***∑_p_ ñ/n_+_*** |  |  |  | ***∑_p_ 1/ñ_p_*** |
| ***ñ_d_*** | 595 | 633 | 594 | 616 | 590 | 474 | 460 |  | **= 3962** |  | **= 5.56** |  |  |  | **= 193** |
| ***ñ*** | 354025 | 400689 | 352836 | 379456 | 348100 | 224676 | 211600 |  |  |  |  |  |  |  |  |
| ***ñ/n_+_*** | 89.4 | 101.1 | 89.1 | 95.8 | 87.9 | 56.7 | 53.4 |  |  |  |  |  |  |  |  |
| **X̄*_d_*** | 30.4 | 32.6 | 32.4 | 31.9 | 34.7 | 25.8 | 25.7 |  |  |  |  |  |  |  |  |
| **X̄** | 924.5 | 1066.0 | 1047.3 | 1018.1 | 1202.4 | 666.7 | 660.9 |  |  |  |  |  |  |  |  |

*n_p_ =* number of participants (788)

*n_d_ =* number of days (7)

*ñ_p_ =* number of days with acceptable data for each participant

*ñ_d_* = number of participants with acceptable data for each day

*n_+_* = days with acceptable data (3962)

*r_p_* = ∑_d_ ñ/*n_+_* = 573

*r_d_* = ∑_p_ ñ/*n_+_* = 5.56

λ*_p_* = [(*n_+_* - *r_p_*) / (*n_+_* - *n_p_*)] = [(3962 – 573) / (3962 – 788)] = 1.07

λ*_d_* = [(*n_+_* - *r_d_*) / (*n_+_* - *n_d_*)] = [(3962 – 5.56) / (3962 – 7)] = 1.00

*n̈_d_* = harmonic mean of *ñ_p_* = [1/*n_p_* ∑_p_ (1/*ñ_p_*)]^-1^ = [1/788 (193)]^-1^ = 4.08
