# Supplementary material for: Reporting the Reliability of Accelerometer Data with and without Missing Values
Source: PLoS One. 2014 Dec 5;9(12):e114402. doi: 10.1371/journal.pone.0114402 (PMC4257690; doi:10.1371/journal.pone.0114402)
Supplement: Table S2 — Variance component equations and estimates. (DOCX) [file pone.0114402.s002.docx]

**Table S2.**

**Variance component equations and estimates for a participant (p) × day (d) design with missing data.**

| **Variance component equations** | | |  |
| --- | --- | --- | --- |
|  |  |  |  |
| **Effect** | ***df*** | **T terms** | **Variance component equations (ô^2^)** |
|  |  |  |  |
| *p* | *n_p_* - 1 | *∑_p_ ñ_p_* X̄ | *((T (pd) - T (d)) / (n_+_ - n_d_)) - ô^2^ pd* |
| *d* | *n_d_* - 1 | *∑_d_ ñ_d_* X̄ | *((T (pd) - T (p)) / (n_+_ - n_p_)) - ô^2^ pd* |
| *pd* | *n_+_* - *n_p_* - *n_d_* + 1 | *∑_p_ ∑_d_ X* | *(λ (T (pd) - T(p)) + λ_d_ (T (pd) - T(d)) – (T (pd) – T(μ))) / (n_+_ - r_p_ - r_d_ + 1)* |
| *μ* | 1 | *n_+_* X̄*^2^* |  |
|  |  |  |  |
| **Actual variance component estimates from 9 yr olds (10-hr wear time criteria)** | | | |
|  |  |  |  |
| **Effect** | ***df*** | **T terms** | **Variance component estimates (ô^2^)** |
|  |  |  |  |
| *p* | 787 | 4510739 | ((5247916 - 3803548) / (3962 - 7)) - 221.8 = 143.4 ^a^ |
| *d* | 6 | 3803548 | ((5247916 - 4510739) / (3962 - 788)) - 221.8 = 10.5 ^a^ |
| *pd* | 3168 | 5247916 | (1.07(5247916 – 4510739) + 1(5247916 – 3803548) – (5247916 – 3766549)) / (3962 - 573 - 5.56 + 1) = 221.8 ^a^ |
| *μ* | 1 | 3766549 |  |

*df*, degrees of freedom; *n_d_*, number of days; *ñ_d_*, participants with acceptable data for each day; *n_p_*, number of participants; *ñ_p_*, days with acceptable data for each participant; *n_+_*, days with acceptable data.

^a^ Total variance is derived by summing participant, day, and participant × day interaction components together.
